# Unveiling the Foramen of Winslow: A Case Report of Internal Hernia and Its Surgical Implications

**DOI:** 10.7759/cureus.90562

**Published:** 2025-08-20

**Authors:** Anirudh Krishnan, Thomas G Mackay, Krishnan Iyengar, Abraham Joel

**Affiliations:** 1 General Surgery, Princess Alexandra Hospital, Brisbane, AUS; 2 Anatomical Pathology, Royal Brisbane and Women's Hospital, Brisbane, AUS; 3 Upper Gastrointestinal Surgery, Princess Alexandra Hospital, Brisbane, AUS

**Keywords:** closed-loop small bowel obstruction, emergency diagnostic laparoscopy, epiploic foramen pexy, foramen of winslow herniation, hernia

## Abstract

A foramen of Winslow hernia is a rare variant of an internal hernia. It is a difficult condition to diagnose with a non-specific history and examination and requires the consideration of both clinical and radiologic features. In this study, we report the case of a 67-year-old Vietnamese woman who presented with abdominal pain and vomiting; a computed tomography (CT) scan depicted small bowel obstruction (SBO) with a transition point within the foramen of Winslow. The patient was taken for an emergent laparoscopic reduction of a hernia, without requiring bowel resection or conversion to open surgery. She recovered well post-operatively with the gradual introduction of diet and the cessation of nasogastric tube (NGT) decompression. In keeping with the existing literature on this rare condition, we conclude that early diagnosis and prompt surgical intervention are necessary to limit morbidity and that further research is required regarding techniques to prevent recurrence.

## Introduction

An internal hernia occurs when abdominal viscera, most commonly the small or large intestine, protrude through a mesenteric or peritoneal defect inside the abdomen. These defects can be physiologic or pathological. Internal hernias form a minority of the subtypes of abdominal hernias, quoted in literature as making up between 0.5% and 1% of all cases [1-3]. Of this, hernias through the epiploic foramen (of Winslow) are exceedingly rare, seen in less than 8% of all internal hernias [2-4]. The epiploic foramen is a potential aperture connecting the lesser and greater peritoneal sacs, bounded by the free border of the hepatoduodenal ligament anteriorly, the inferior vena cava posteriorly, the caudate lobe of the liver superiorly, and the first part of the duodenum and common hepatic artery inferiorly. Approximately 200 cases are documented in the medical literature regarding epiploic foraminal hernias, with a 2.5 times greater incidence in men compared to women [3-5]. This condition presents a mortality rate as high as 49% in previous case studies, often due to a lack of early diagnosis, clinical suspicion, and/or early surgical intervention. The documented high morbidity and mortality are attributed to its frequently non-specific presentation and the absence of classic radiographic features of hernia and bowel obstruction [1,3,6]. We present a case of acute small bowel obstruction (SBO) secondary to an internal hernia through the epiploic foramen (of Winslow), requiring emergency surgical reduction and pexy of the epiploic foramen.

## Case presentation

A 67-year-old Vietnamese woman with a history of gastroesophageal reflux and well-controlled hypertension presented to a tertiary healthcare facility with a nine-hour history of sudden-onset right upper quadrant abdominal pain. The pain began shortly after a meal and was associated with nausea and vomiting and radiated to her back. There were no obstipation and no recent changes to bowel habits, dysuria, nor history of abdominal surgeries or trauma. System review and supplementary history were unremarkable. The patient's observations on arrival to the emergency department (ED) were stable with a heart rate of 75 beats per minute (bpm), a blood pressure of 143/88 mmHg, oxygen saturations of 98% on room air, and a respiratory rate of 20 breaths/minute. Physical examination demonstrated peripheral signs of dehydration and a soft but distended abdomen that was tender without peritonism over the right side, without any palpable masses or palpable abdominal wall hernia.

**Figure 1:**
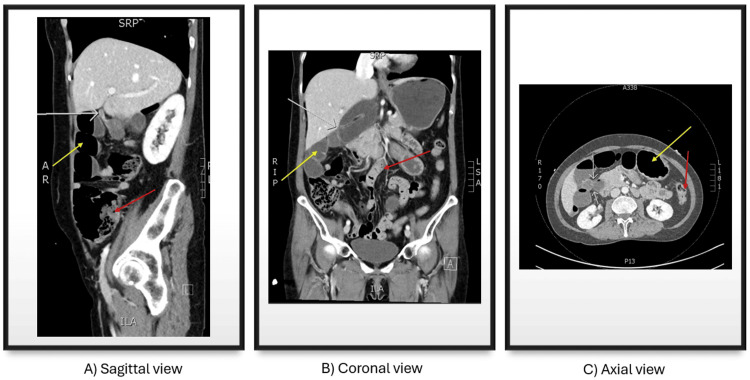
CT scan demonstrating sagittal (A), coronal (B), and axial (C) views of the herniating loop of the small bowel into the epiploic foramen (of Winslow) with abrupt transition point (marked with white arrows), proximal bowel dilatation (marked with yellow arrows), and distal collapsed loops of the bowel (marked with red arrows). CT: computed tomography

Initial blood tests depicted a neutrophilia of 10.7 × 109/L, normal lactate, and otherwise reassuring normal parameters. A plain chest radiograph noted a stomach distended with gas and no free air under the diaphragm. A contrast-enhanced computed tomography (CT) scan of the abdomen subsequently demonstrated a closed-loop small bowel obstruction (SBO) with air-fluid levels and associated mesentery herniating into the epiploic foramen, displacing the portal vein, proximal duodenum, and common bile duct anteriorly, with associated fat stranding (refer to Figure 1). There were no additional features of free fluid, pneumoperitoneum, or perforation.

**Figure 2:**
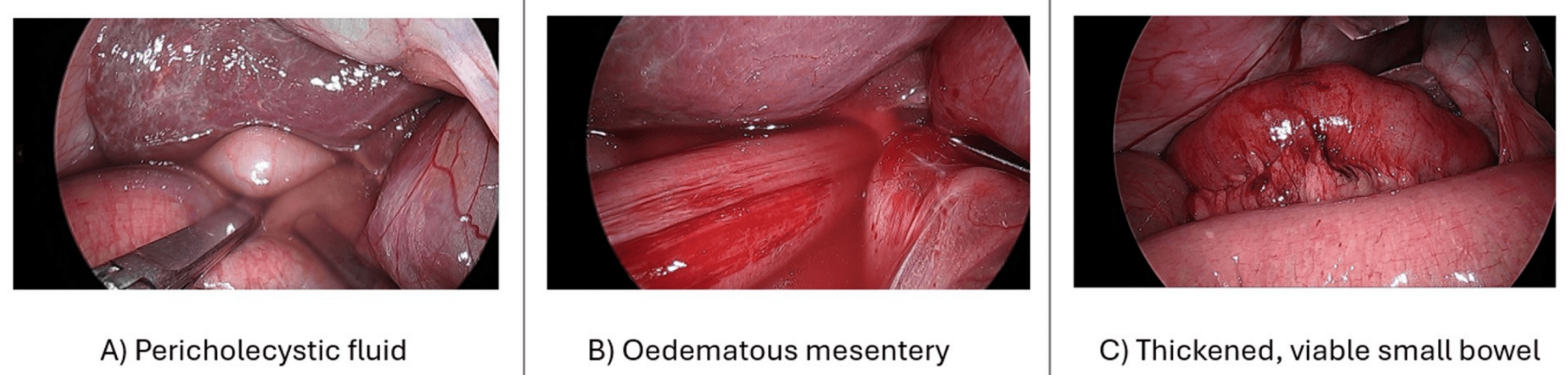
Laparoscopic views of turbid fluid in the subhepatic space (A), the loop of the small bowel extending into the epiploic foramen (B), and edematous but viable small bowel post hernia reduction (C).

Initial management was commenced by making the patient nil by mouth, intravenous (IV) fluid resuscitation, the insertion of a nasogastric tube (NGT) for gastric decompression, an indwelling urinary catheter (IDC), and prepping the patient for emergency laparoscopic surgery. Laparoscopy was conducted within three hours of the patient's review in the ED and approximately 12 hours post symptom onset. Turbid fluid was noted in the paracolic gutters bilaterally immediately on entry, with dilated loops of the small bowel in the upper abdomen. A segment of small bowel looping through the epiploic foramen was demonstrated and successfully reduced with manual traction (refer to Figure 2). The reduced segment of the small bowel was bruised but viable on inspection, and a bowel resection was not required. The small bowel was thoroughly inspected, and the epiploic foramen was partially pexied from the mesogallbladder to the perirenal fascia to loosely close approximately one-third of the foramen to prevent future occurrences.

The patient had an unremarkable journey post-operatively. Her diet was upgraded progressively, and she was discharged from the hospital on the third post-operative day. She was well at her six-week post-operative review in the outpatient clinic and discharged from the general surgery service.

## Discussion

There are numerous predisposing factors that have been postulated for the formation of epiploic foraminal hernias, frequently differentiated into acquired and congenital types. These hernias are rarely noted due to the normal anatomic structure of intra-abdominal viscera and intraperitoneal pressure gradients that prevent the opening of the foramen and the acceptance of herniating structures from the greater sac of the peritoneal cavity. Therefore, it is hypothesized that altered intraperitoneal pressures, excessive visceral mobility, and/or the abnormal enlargement of the epiploic foramen could place patients at higher risk of these hernias occurring [7,8]. Patients with altered anatomy, such as those post cholecystectomies, may have increased visceral mobility with an abnormally long mesentery. This can lead to the enlargement of the right lobe of the liver, acting to direct viscera into the foramen. The above patient, however, had not had previous abdominal surgeries, making this an unlikely etiology. An exact measurement of the foraminal diameter was not conducted during the emergency laparoscopy, but Moris et al. [3] define a foramen of >3 cm (or greater than a one-to-two finger width) as being high risk for the occurrence of hernias [9]. This is an inherited risk factor, though the patient had no documented family history of internal hernias. Finally, acquired risk factors such as pregnancies and colonic tumors increase intraperitoneal pressure and can increase the risk of all internal hernias, including those involving the epiploic foramen [1]. These were not relevant in the case described above; however, another risk factor that is more infrequently mentioned in the literature is overeating [10]. The patient in the present case described symptom onset following a large fatty meal, in keeping with this potential risk factor. The mechanism for this etiology of internal hernias involving the epiploic foramen would need to be reviewed in larger cohort studies or systematic reviews.

The diagnosis of internal herniation is made through a combination of clinical findings, laboratory tests, and imaging. In this case, the abdominal CT provided the most information with regard to the diagnosis, hernial contents, and viability of bowel. This is in keeping with existing literature that CT imaging, especially multidetector CT (MDCT), can provide high-resolution images with a particular focus on the perfusion/viability of the involved viscera [3,9]. The classic radiographic findings described are the findings of mesenteric vessels directly posterior to the portal vein, common bile duct, and hepatic artery but anterior to the inferior vena cava. The radiographic findings described in this case were similar to the above and noted the presence of a transition point leading toward the foramen. Other findings can also include fluid in the lesser sac, the narrowing of the portal vein, and the anterolateral displacement of the stomach [1]. These findings are highly dependent on which viscera herniate through the orifice. Sixty percent of cases note small bowel herniating, while the cecum and ascending colon account for 30%. The remaining minority are rare cases of transverse colon and gallbladder herniation [1-5,10]. The presentation and symptomatology vary depending on the herniating structure, though cross-sectional imaging remains the gold standard for diagnosis. A wide list of differentials should also be considered, including other etiologies of SBO such as adhesions, abdominal wall hernias, malignancy, other sites of internal hernia, and large bowel obstruction with an incompetent ileocecal valve.

The incidence of bowel necrosis in hernias through the epiploic foramen is high in cases where diagnosis is delayed. There appears to be a varied presentation of ischemic viscera in the epiploic foramen, with some cases noting an insidious onset of pain and distension [9,11], while others describe sepsis, hemodynamic instability, bleeding, and liver function derangement [12,13]. In all cases, segments of nonviable bowel were resected, prolonging inpatient stay and post-operative recovery. The absence of bowel resection in the setting of visceral necrosis in this case is a testament to the prompt diagnosis and operative intervention and hernia reduction. Laparoscopy is generally recommended as the modality of choice in cases without established bowel necrosis or hemodynamic instability [3,9-11]. Simple traction is suggested as a first line in the reduction of the hernia and was the only intervention required in the above case. If this is unsuccessful, other techniques in combination with a laparotomy can be deployed, such as opening the lesser sac, large duodenopancreatic detachment (Kocher's mobilization), or opening the gastrohepatic ligament to release the strangulated hernial contents [2,11-14]. The closure of the epiploic foraminal defect with nonabsorbable sutures remains a point of contention and was performed in the above case as a temporizing hernial closure technique. Thus far, there have been no reported cases of recurrent epiploic foramen hernias, and hence, no statistical advantage to closing the foramen is evident [3,12,15]. It is thought that the foramen typically obliterates in the post-operative healing course with fibrotic replacement and peritoneal adhesions. This is combined with an added risk of injury to the hepatic artery, bile ducts, and portal vein thrombosis when conducting the primary closure of the foramen, with some studies suggesting avoiding active closure when possible. The decision to be made regarding closing the foramen, thus, remains unclear [2,3,11,14]. Larger cohort studies and reviews should look to classify the hernia types by their predisposing risk factors to help guide the appropriate preventative interventions and consider the rates of injury to critical structures when attempting the closure of this defect.

## Conclusions

Closed-loop obstruction secondary to internal herniation through the epiploic foramen is a rare presentation and has the potential to result in significant patient morbidity. Thus, it remains an important differential diagnosis for the radiologic interpretation of the acute abdomen and is best managed with prompt operative intervention for optimal outcomes. Surgeons should remain cognizant of this presentation due to its potentially wide symptomatology and the common surgical techniques deployed in these cases. Further research into this condition should look to clarify the relevance of preventative measures in these cases and the various acquired risk factors for the development of this rare condition.
